# Measuring Outcomes in Evaluations of Interventions to Prevent Youth Crime and Violence: Challenges and Potential Solutions

**DOI:** 10.1007/s10935-026-00921-x

**Published:** 2026-07-01

**Authors:** Sajid Humayun, Finlay Green, Helen Downham, Nick Axford

**Affiliations:** 1 Institute of Lifecourse Development, University of Greenwich, London, UK; 2 Dartington Service Design Lab, Buckfastleigh, UK; 3 University of Plymouth, Plymouth, UK

**Keywords:** Crime, Evaluation, Measurement, Outcomes, Violence, Youth

## Abstract

There is a clear imperative to prevent youth crime and violence owing to the significant social and economic costs for individuals involved and wider society. A considerable evidence base for ‘what works’ in this space exists but gaps remain. To address these, it is necessary to evaluate intervention effectiveness. The aim of this article is to explore challenges and potential solutions regarding outcome measurement in such evaluations. To do this we draw on the literature and our experience as evaluators. We argue that outcome selection should be informed by a robust theory of change, and that normally this will mean focusing on non-crime/violence outcomes. We identify challenges with self-report measures of youth crime and violence, and consequent effects on participants’ engagement in the intervention and the quantity and quality of data they provide. We also discuss ways of using administrative data on crime and violence in evaluations and the practical, scientific and ethical issues these raise. While the challenges identified can partially be addressed, we conclude that currently evaluations of preventive interventions should mostly focus on intermediate outcomes with a known causal link to crime and violence. The longer-term impact on crime and violence can be tracked using administrative data accessed via an archive. We appreciate policymaker and funder pressure to quantify short-term intervention impact on crime and violence but contend that it is important not to force the issue. Meanwhile, it is essential to test the feasibility and acceptability of alternative approaches to measuring crime and violence in evaluations and to share the learning.

## Introduction

In the year ending March 2023, around 34,300 offences were committed by children and youth (CYP) aged 10–17 years in England and Wales that resulted in a police caution (formal out-of-court warning) or sentence at court (Youth Justice Board [YJB], 2024a).^1^ While this number is 65% lower than 10 years earlier and reflects decreases this century in other countries in Europe but also North America, Australia and New Zealand (Polglase & Lambie, 2024), it remains a significant issue for society, with some evidence that reductions in low to moderate offending have masked increases in chronic offending (McCarthy, 2021). Violence against the person offences in England and Wales saw the largest increase in proportion over that period, from 21% to 34% (YJB, 2024a). Some young people are disproportionately affected, notably boys, CYP from Black backgrounds and those living in the poorest areas (Youth Endowment Fund [YEF], 2024).

The economic and social costs of youth crime and violence are well established. One analysis, for instance, calculated that in the preceding 11 years serious youth violence across England and Wales had a total economic and social cost of £11 billion (Irwin-Rogers et al., 2020). This covered the police and criminal justice system, health and victim services, and costs associated with physical and emotional harm and lost economic output. There is a clear imperative to act to prevent and reduce youth crime and violence.

A series of school-, family- and community-based psychosocial interventions to prevent and address youth crime and violence have been developed and evaluated in the last 25 years. These include social-emotional and bullying prevention programmes, youth mentoring, parenting support and various types of individual or family-orientated therapies (Fagan & Catalano, 2013; Farrington et al., 2017; Kovalenko et al., 2022). However, gaps in the evidence base remain. Efforts to fill these continue apace; in England and Wales, for example, the Youth Endowment Fund (YEF) – a government ‘What Works’ centre established in 2019 with a £200 million endowment and 10-year mandate from the Home Office to develop and implement knowledge on preventing youth violence – invests heavily in efficacy and effectiveness trials.

Ultimately, the task of determining the effectiveness of psychosocial interventions to prevent youth crime and violence requires demonstrating a causal link between the intervention and crime and violence outcomes. By crime and violence we mean criminal/offending behaviour, serious/aggressive anti-social behaviour, violence towards others and cyber/online violence (Ullman et al., 2024)^2^. Detecting this link in intervention effectiveness evaluation studies, notably randomised controlled trials (RCTs) or quasi-experimental designs, is challenging. Drawing on our experience as evaluators, and the wider literature, we aim in this article to (i) articulate why it is often difficult to measure these outcomes meaningfully in evaluations and (ii) discuss potential solutions and associated challenges. Our arguments are informed in particular by our involvement in YEF-funded evaluations but are by no means limited to a single funder or its portfolio of studies. The article is essentially in two parts. The first focuses on the selection of relevant outcomes, while the second focuses specifically on measuring crime and violence outcomes.

### Selecting Relevant Outcomes

Studies can only test the effectiveness of interventions if they measure the outcomes targeted by those interventions. A key aim of feasibility work therefore needs to be identifying viable outcome measures. However, intervention approaches at an early stage can often struggle to identify their primary outcome, even if they have been delivered for some years. There are arguably three reasons for this.

First, many delivery organisations are not well placed to collect robust outcome measures and may have been using minimal or ad hoc outcome monitoring approaches. This may be due to the view that such measures do not capture the specific problems that CYP and families face or that they are not sensitive to change (Forrester, 2017). There are also challenges around participant burden, comparability across different services settings and, for some measures, financial cost (Deighton et al., 2014).

Second, even when psychometrically sound measures have been used, they may be broad in terms of outcomes covered. For example, many providers use the Strengths and Difficulties Questionnaire (SDQ; Goodman, 1997). This is probably the most widely used CYP mental health questionnaire in the UK (Wolpert et al., 2015), with subscales for conduct problems but also hyperactivity, internalising problems, peer problems and prosocial behaviour. Its widespread use is beneficial when pooling effects across different studies. However, the total score is often used as a measure of clinical change in evaluations. This can lead developers to think that their intervention is designed to improve a wide range of outcomes rather than targeting a particular problem. This is unlikely to be the case.

Third, often a clear theory of change (ToC) has not been properly developed. In a survey of 113 YEF grantees, for instance, 69% lacked a ToC for their intervention at the point of funding (Simpson et al., 2021). Intervention approaches can develop out of adaptations to routine practice by clinicians and other practitioners who may feel that they are achieving positive change with CYP without having formally evaluated treatment effects, sometimes due to resistance to outcome-focused evidence-based interventions (Lilienfeld et al., 2013). However, further into delivery it may be deemed necessary to formally evaluate the intervention to acquire further funding, despite key mechanisms not being clearly specified (Simpson et al., 2021; Bierman et al., 2023). This might be because the intervention is believed to have a range of positive outcomes but, if so, these are likely secondary effects to the key outcome it is targeting. For example, a family therapy approach may succeed in reducing internalising problems even when it is primarily designed to reduce substance misuse and externalising problems (e.g., Schaub et al., 2014).

The lack of a clear ToC can make it challenging to identify the most appropriate outcomes to measure. An intervention to reduce youth violence might have quite context-specific effects, for example reducing violence at school but not at home, or reducing bullying but not reactive violence. Furthermore, the lack of a ToC makes it impossible to identify likely mediators of treatment effect. For example, an intervention working with CYP to reduce aggression might improve that outcome by improving anger regulation (Coping Power; Lochman & Wells, 2004) or by improving social cognition and perspective taking (PATHS; Greenberg et al., 2004). Similarly, a family-based intervention such as Functional Family Therapy (FFT; Alexander & Parsons, 1973) might improve CYP outcomes by changing parent behaviour but, equally, it might be changes to CYP behaviour that must come first. These examples involve quite distinct mediators of treatment effect and identifying and measuring them is often crucial to refining and improving interventions.

Often in evaluations of youth crime and violence prevention interventions there is pressure to measure effectiveness in terms of crime and violence. Understandably, policymakers and funders want to invest in interventions that impact on ‘big’ outcomes with major health and economic implications for participants and wider society. However, it is not always right or possible, at least immediately. Preventive interventions are less likely than more targeted treatments to impact significantly on crime and violence in the short-term. Rather, they are designed – and more likely – to affect proximal or intermediate outcomes, or mediators of crime and violence, in the knowledge that doing so may reasonably be expected to prevent or reduce crime and violence in the medium- to long-term.

It is important to ensure that the proximal outcomes are demonstrably causal risk factors for offending and not purely correlational. Fortunately, a wealth of data exists on causal risk factors of crime derived from longitudinal studies (mostly from the US). These studies have identified key modifiable risk and protective factors (or needs and strengths) at multiple levels of the ecological model that influence or are associated with youth crime and violence (e.g., Ayano et al., 2024; Ullman et al., 2024). Thus, there is value for science and policy in an evaluation demonstrating that a preventive intervention reduces substance use (Assink et al., 2015) or enhances secure attachment (Hoeve et al., 2012), parenting skills (Flanagan et al., 2019) and prosocial attitudes and values (Scott & Brown, 2018), without needing to show *in that evaluation* that it reduces crime or violence. Another consideration is the extent to which changing one or two proximal outcomes is likely to lead to a reduction in offending when CYP may have a constellation of risk factors. Our view would be that this is an issue for the intervention ToC to resolve. The purpose of measuring outcomes is to test for treatment effects. No individual intervention, even a multi-modal one, will target all potential causal factors, so it would seem sensible for evaluations to focus on the specific outcomes that the intervention is trying to change.

The argument to focus on proximal outcomes is made more forcibly by advocates of the Child First or Positive Youth Justice models, which emphasise promoting positive CYP outcomes generally with desistance from crime as a specific secondary outcome – albeit with the former theorised to lead to the latter (Hazel & Case, 2024). Further, and as the next section shows, there are good practical and ethical reasons for not always seeking to measure crime or violence in evaluations, especially for more preventive interventions. Of course, this argument strengthens the case for measuring the longer-term impact of preventive interventions, which is traditionally neglected.

### Measuring Crime and Violence Outcomes

Here we discuss options for measuring youth crime and violence outcomes in evaluations, and their associated strengths and challenges (see Table 1 for an overview). We address self-report measures first, followed by administrative data.

**Table 1:** Approaches to measuring crime and violence outcomes

Outcome measurement approach	Strengths	Challenges
*Self-report measures*		
1. Self-report measures of crime/violence administered to CYP pre- and post-intervention	• Some measures demonstrate good psychometric properties and practical usability • Can yield more timely data than using official records • More measurement sensitivity than official records; explore a wider range of behaviours and provide a more detailed assessment of their frequency • Have been found to provide accurate estimates of prevalence and mean offending frequency (fewer adverse consequences of disclosing offences)	• General resistance among some practitioners and young people to self-completion measures • Some CYP may feel stereotyped, stigmatised or discriminated against by crime/violence questions • This can tarnish CYP relationship with practitioners and reduce their engagement in the intervention, which in turn may impair effectiveness • It can also contribute to dishonest or incomplete question responses (i.e., it adversely affects data quantity and quality) • Individuals with police-recorded offences are less likely to participate in observational studies • Susceptibility to socially desirable responding and inaccurate recall
2. Computer Assisted Personal Interviewing (CAPI) or other computerised self-report administration of measures (i.e., a different way of administering the same measures as in the previous approach)	• Increased disclosure of offending i.e., more honest question responses	• May have the opposite effect to that intended and reduce disclosure because CYP fear that honest answers will trigger safeguarding or crime reports by researchers (this could be mitigated if a mechanism is put in place to ensure researchers cannot access responses until the project ends) • May fail to mitigate against missing data due to participants skipping questions compared to researcher administered questionnaires
3. Post-test only design (i.e., no pre-test)	• Averts the potential damage to CYP engagement in the intervention caused by asking baseline questions about crime/violence • Responses are not influenced by a pre-test • Can be used when a pre-test is not possible	• Precludes tests for baseline equivalence • Hard to detect and control for attrition bias • Unable to control in analysis for pre-test measurements
4. Transition questions at the end of the intervention asking CYP whether and by how much their participation in crime/violence has changed compared with when the intervention started	• Averts the potential damage to CYP engagement in the intervention caused by asking baseline questions about crime/violence	• Subjective i.e., participant perspective • Relies on recall over an extended period, so may be inaccurate • Untested in evaluations of youth crime/violence prevention interventions (to our knowledge)
5. Retrospective pre-test, whereby respondents report *after* (rather than before) an intervention on knowledge, attitudes or behaviours before the intervention	• Averts the potential damage to CYP engagement in the intervention caused by asking baseline questions about crime/violence • Helps to address response-shift bias	• Introduces other biases, for example because participants feel the need to justify the effort invested in the intervention, so inflating effect sizes
6. Ask about likelihood of engaging in crime or violence in the future	• Avoids asking CYP questions about past behaviour which they may not want to answer	• Unclear if it actually measures offending
*Administrative data*		
7. Access crime/violence data on study participants held by official agencies *during the course of the research*	• Saves time and money involved in gathering primary data • Avoids acceptability issues for practitioners and CYP participants associated with self-completion measures • Provides a more holistic view of outcomes • May capture offences committed by individuals who do not participate in observational studies	• Data can be hard to access and/or inconsistent • Delays in obtaining data may extend beyond the project endpoint, preventing its use • Data can be partial, complex and biased (e.g., underreporting of offences, over-policing some communities or minority ethnic groups), so risks being misinterpreted • Participants may not consent to its use, or do so with a limited (false) appreciation of what is involved • May be less pertinent for preventive interventions focused on intermediate outcomes (i.e., not part of their ToC, CYP less likely to have contact with the justice system)
8. Facilitate access to crime/violence data on study participants held by official agencies *in the future* (i.e., beyond the end of the study), for example the YEF archive	• Creates the opportunity for researchers to track crime/violence outcomes in the long-term, which is when they are more relevant • Aligns with the typical ToCs of preventive interventions, which posit crime/violence as distal outcomes • CYP (especially those who were young in the original study) are more likely to have had contact with the criminal justice system • Frees evaluators to focus on measuring known mediators of crime/violence, knowing that the impact on crime/violence outcomes can be examined later	• Participants may not provide consent or assent, particularly as the complex processes involved in depositing and accessing data are difficult to explain simply to participants • Issues with statistical power if original study was not sufficiently powered (variables in archive likely measure events that are rare) • Challenges in identifying and destroying data if participants subsequently choose to withdraw from a study (because of the pseudonymisation process involved in data linkage) • Builds in a long delay to obtaining offending outcomes, when policy-makers want evidence on effectiveness promptly

#### Self-report Measures

CYP self-report measures are used widely in evaluation and practice, with some measures demonstrating good psychometric properties and practical usability (Deighton et al., 2014). In principle, this approach has significant benefits when measuring youth offending. Compared with using official records, which can be complex and time-consuming, it can yield more timely data. It has more measurement sensitivity than official records, which represent the ‘tip of the iceberg’ in terms of behaviour. For example, longitudinal studies in the US and UK show that official convictions tend to underestimate the true rate of crime significantly (Basto-Pereira & Farrington, 2020). Similarly, a comparison of self-report and police data in one UK study found that the prevalence of self-reported violence was much higher than rates according to police records (Cornish et al., 2024). This is likely because a lot of violent offences are not reported to the police, and there are fewer consequences from disclosing crime or violence perpetration in a self-report measure. Furthermore, missing data in police records can be due to poor data recording practices (O’Connor et al., 2022) and whilst some solutions exist to maximise validity (Spence & Crivatu, 2025), these are limited. Self-report measures can explore a wider range of behaviours and provide a more detailed assessment of their frequency than police records, which are biased by limited reporting of crime and less likely to capture offending in some geographical areas (Buil-Gil et al., 2021). Additionally, self-report measures have been found to provide accurate estimates of prevalence and mean offending frequency (Thornberry & Krohn, 2000), although this varies by recall period and ethnicity (Li, 2026).

One such measure is the 19-item Self-Report Delinquency Scale (SRDS), which covers a range of antisocial and offending behaviours and has been validated for 10–17-year-olds. It produces two scores: *variety* – the number of different offending behaviours the respondent reports involvement in; and *volume* – the estimated minimum total number of offending behaviours committed. The measure correlates with official police arrests (McAra & McVie, 2005), while reported internal consistency is 0.87–0.92 with an inter-item correlation of 0.19 (Humayun et al., 2017; Fonagy et al., 2018). The YEF encourages its use in YEF-funded efficacy and effectiveness evaluations to maximise learning across projects. The SRDS was first developed and used in a longitudinal study of approximately 4,000 CYP starting their first year of secondary school in Edinburgh, Scotland, in 1998.

There are challenges with self-report measures, however. One is that observational studies underestimate crime and violence because individuals with police-recorded violence are less likely to participate in such studies – also known as ‘participation bias’ (Cornish et al., 2024). Other biases include a susceptibility to socially desirable responding and inaccurate recall, while some youth and practitioners also report general qualms about self-report measures (de St Croix & Doherty, 2024).

Moving to the more specific, at least three YEF trials have encountered firm resistance to the SRDS. In a pilot study of the school-based youth mentoring programme Becoming a Man (BAM), managers and practitioners felt that it would stigmatise CYP participation and undermine the practitioner-CYP relationship, thereby impairing programme effectiveness (Green et al., 2026). There was also a perceived risk of reinforcing negative racial stereotypes about Black adolescent boys in a community with longstanding issues of institutional racism and criminalisation. A workshop with CYP who shared key characteristics with BAM participants reinforced these concerns. Participants highlighted distrust of authority and white professionals, and suggested that such measures were racially charged and indicative of assumed criminality, and that they would lead to dishonest responses and feelings of discrimination. Consequently, it was agreed with the YEF not to use the SRDS. There was a similar response in a YEF-funded pilot trial of a CBT-informed intervention (Cattan et al., 2023), where practitioners felt that the SRDS was not aligned with Child First principles and was likely to cause harm to CYP. These issues may reflect in part use of the SRDS in a different scientific context to its original use. In the Edinburgh study, it was completed at population level as one of several measures; it was not designed to measure intervention effects.

Where self-report measures such as the SRDS are used, the quantity and quality of the data collected can be poor. In a pilot trial of Multisystemic Therapy (Langdon et al., 2023) only eight of 41 CYP completed all assessments, while in an FFT pilot (Humayun et al., 2023) 80% of CYP claimed no engagement in any criminal activity except truancy or fare evasion, despite case records suggesting otherwise. Challenges with self-report measures may be exacerbated for specific populations. These two examples were trials for Child Criminal Exploitation (CCE), where CYP are often threatened by gangs or organised criminal groups not to disclose their activities to authorities (Coliandris, 2015; Robinson et al., 2019). They are unlikely to distinguish between a social worker and a researcher requesting data. It is worth noting that some (but not all) of these studies were affected by the COVID-19 pandemic, which may limit the generalisability of some lessons learned about the SRDS. Furthermore, there appear to be better completion rates with the SRDS in trials with higher risk samples, notably CYP in police custody (Coulton et al., 2023), those who have committed violent offences or displayed violent behaviour (Bandyopadhyay et al., 2024a; although only at baseline), or with associations with peers or family members involved in serious violence or organised crime (Clements et al., 2023; although there was high attrition in this trial).

One potential solution to increase the validity of self-report measures is to have practitioners, rather than researchers, administer self-report measures because they are more likely to have a trusted relationship with CYP. However, this may impair the practitioner-CYP relationship by making CYP feel stigmatised. Further, it might introduce bias and increase missing data rates. For instance, a recent evaluation of a programme to address child-to-parent violence effectively failed because practitioners were tasked with collecting the primary outcome data (SDQ) and most of it was missing (less than 15% CYP data completed; Adler et al., 2023). The authors concluded that this may have been due to practitioners feeling the assessment was too much of a burden for families, although families did not mention this as a problem. Early feasibility work should assess whether a reasonable number of CYP can be recruited when a full assessment is included, and this should be included in stop-go criteria (Avery et al., 2017). This needs to be balanced with the risk of failing to capture important data in pursuit of using the smallest possible assessment battery.

The lack of acceptability of the original SRDS (McAra & McVie, 2005) among CYP and practitioners may partly be attributed to arguably outdated and pejorative language. Use of the more recent International Self-Report Delinquency measure (Marshall et al., 2022) appears to improve completion rates compared to the original measure (Bandyopadhya et al., 2024b). It has also been used in a number of different countries and demonstrated reasonable convergence with official records (Enzmann et al., 2010), although this has also encountered resistance in evaluation studies (Mehay et al., 2024). Another potential method to improve disclosure is Computer Assisted Personal Interviewing or other computerised self-report administration of measures. While interviewer-administered measures may mitigate against missing data due to participants skipping questions, there is some evidence that collecting offending data using a computerised self-report method results in greater reporting of offending (Gomes et al., 2024). However, when questionnaires include items about behaviours that may trigger a safeguarding response from researchers, such as CCE or child sexual abuse (CSE), it is necessary to include a statement on breach of confidentiality and the possibility that answers might be shared with relevant agencies. This is likely to reduce reporting of these items and other offending questions, irrespective of administration mode. This problem, long recognised in child maltreatment research (Knight et al., 2000), has received less attention in youth offending studies.

A solution to the disclosure problem, currently being trialled in efficacy trials of FFT for CCE (Humayun et al., 2024) and the Strengthening Families, Strengthening Communities parenting programme for youth offending and violence (Humayun et al., 2026), involves including measures of offending, CCE, CSE and substance misuse in a computerised self-report survey that is inaccessible to researchers until the trial ends, and to inform CYP of this before asking them to complete measures. This part of the assessment is stored separately and maintained by an independent researcher who has no identifiable CYP information and therefore cannot breach confidentiality. CYP are thereby assured that no action can be taken based on their responses (but are given contact details if they want further support). The hope is that this method will improve reporting on these measures but it will not be possible to assess its success until 2027 when trial data collection ends. A more radical alternative is to administer self-report measures anonymously, although we are unaware of this approach being trialled in practice. The data would allow for testing group differences but significant downsides include not being able to track individual change, undertake moderator or mediator analyses or covary for baseline outcome scores. An alternative solution to improve disclosure involves asking about longer periods of time rather than recent offending (Gomes et al., 2023) but this is often not viable in evaluation studies.

There are several ways of addressing the problem of pre-test measures interfering with rapport-building between the CYP and a practitioner when an intervention starts. Most simply avoid an upfront pre-test measure. One is to use a post-test only design, which means there is no baseline to compare against. This has other advantages; responses are not influenced by a pre-test and it can also be used when a pre-test is not possible. However, it brings disadvantages, such as precluding tests for baseline equivalence, making it hard to detect and control for attrition bias, and not being able to control in analysis for pre-test measurements.

Self-report ‘transition questions’ when an intervention ends offer another alternative. These ask respondents whether and by how much something in their life has changed compared with an earlier time point and derive from clinical methods for analysing change in patient health and quality of life (e.g., Ward et al., 2015). For example, an anchor statement “Compared with October 2024, when you started [intervention]…” would be followed by a domain-specific question, such as “…, do you commit crimes involving property (e.g., stealing things that don’t belong to you)?” and then three response options: “More, About the same, Less”. Participants selecting “More” or “Less” would be asked a further question to indicate degree, such as “By how much?”, with several response options, for instance “Hardly at all, A little but noticeable, A moderate amount, A lot”. We have not seen this approach in evaluations of youth crime and violence interventions, so it would need to be tested. Obvious challenges include its subjective nature and dependence on participants’ recall over an extended period.

A variation of this is the retrospective pretest, where respondents are asked to report post-intervention on knowledge, attitudes or behaviours pre-intervention (Little et al., 2020). It is primarily intended to address ‘response-shift’ bias, whereby intervention participants recalibrate their prior understanding of intervention content such that post-test scores are worse than pre-test scores even though improvements in the outcome have occurred. For example, a participant may report greater levels of violence post-test than pre-test not because they have become more violent (they may have become less violent) but because their understanding of what constitutes violence has changed. However, in addressing one possible bias, the retrospective pretest risks introducing other biases, meaning that caution is advised when using it, especially when seeking to make causal claims (Hill et al., 2020). For example, respondents might feel a need to justify the time and effort invested in the intervention or want to appear to oneself or the evaluator to have improved over time, leading them to inflate the difference between post-test and retrospective pre-test.

A novel approach trialled in the Your Choice study (Cattan et al., 2023) involved developing a new measure that asked about participants’ likelihood of engaging in criminal behaviour over the next month rather than asking about previous criminal behaviour. The internal reliability of the new scale was good and it correlated with SDQ conduct problems. However, only two-thirds of randomised CYP completed the baseline survey (post-intervention survey completion rates forthcoming), and it is unclear whether the scale actually measures offending. CYP perceptions of the likelihood of engaging in offending may not be predictive of their actual offending behaviour, possibly because offending is strongly associated with impulsivity (Maneiro et al., 2017). Therefore, an intervention might impact intentions to offend without affecting actual offending.

#### Administrative Data

An alternative or complement to self-report measures is outcome data drawn from administrative systems. This approach can save money and time by reducing primary data collection for researchers (Administrative Data Research UK, 2025) and avoid acceptability issues for practitioners or CYP associated with self-report measures. It may also provide unique insight into populations and how they interact with systems across different sectors (Administrative Data Research UK, 2025). For example, the national evaluation in England of the Troubled combined datasets from the Ministry of Justice (MoJ), Department for Education and Department for Work and Pensions to provide a holistic review that would have been impossible using primary data collection (Man & Taylor, 2019). Thus, analyses were able to examine data on adult and youth offending (custodial sentences, convictions, cautions), working-age adults claiming out-of-work benefits, and children needing help from children’s services (including being in care). Another advantage of official records is that they may capture offences committed by individuals who do not participate in observational studies.

However, caution should be exercised. In terms of accuracy, biases in policing practice can adversely affect official records. Thus, a greater concentration on more deprived communities, or high stop and search rates for some minority ethnic groups, can increase the likelihood of certain individuals being arrested (Cornish et al., 2024). More practically, administrative data in youth justice is held across a variety of systems, making it difficult for researchers to interpret (YJB, 2025). There have been efforts in England and Wales to standardise (YJB, 2024b), which should help researchers using youth justice data. This guidance includes a requirement to use AssetPlus as the assessment and planning framework for all statutory cases in the community and secure estate. AssetPlus aims to provide a central record for each CYP (YJB, 2014). It includes sections covering a CYP’s history, current activity and future planning. However, not all CYP data relevant to youth crime research are covered. For example, AssetPlus is not mandatory for CYP under out-of-court disposals (YJB, 2024b), which covers a large portion of CYP supported by youth justice services who are at risk of offending or managed outside formal court processes (YJB, 2023). This highlights the complexity of youth justice data and the necessity of researchers fully investigating what is covered within datasets. This also includes how data are collected, noting the potential for human or system errors. For example, ethnicity as recorded on the Police National Computer (PNC) is visually identified by police officers, not self-identified, and does not include a ‘Mixed’ ethnicity group (YJB, 2025). Innovative analysis techniques can be explored to mitigate inconsistencies between datasets, such as probabilistic matching recently used by the MoJ to explore repeat offending of CYP (MoJ, 2025).

Administrative systems are primarily case management systems and not typically designed to measure outcomes systematically. For example, in a recent trial of FFT for CCE (Humayun et al., 2023), it was assumed that a measure of CCE and/or county line drug network^3^ involvement would be captured for all CYP on social care systems, because this is why they had been referred. This was not the case, however, because of technical limitations on how risks are recorded on these systems. Reports derived from UK social care case management systems often use a flag system whereby the most pressing risk to the CYP is identified but this can be changed based on subsequent assessment or other data. If a CYP is identified as being involved in gang activity or being criminally exploited, this can be overwritten and lost if, for example, a concern about sexual abuse is identified. The alternative is to rely on case notes, where the original risk might be retained, but this is very time consuming and is again completely reliant on individual practitioners completing these fully (Allnatt et al., 2022).

Data collected by other agencies may also be hard for evaluators to access. In the case of BAM, it was agreed to explore using PNC data as an alternative to the SRDS. However, the process for obtaining such data was initially opaque and forecast delays (up to 18 months) in obtaining data would have made it impossible to report findings within the project timeline (Green et al., 2025). Challenges included the lack of a clearly marked ‘front door’ to access data, the need to complete a detailed data request form followed by lengthy correspondence with data holders to clarify points, and a lack of written guidance about the meaning of the many variables (often with confusing names or codes). Similar barriers and delays were experienced in the first UK RCT of FFT (Humayun et al., 2017). While PNC data was acquired, this took many months and involved a series of meetings with Home Office officials to negotiate. Helpfully, a new YEF guide for evaluators, designed to alleviate these problems, describes various relevant administrative datasets, such as local police and PNC data, how to access and use them and their strengths and weaknesses (Ellison & Cook, 2024).

A further challenge for administrative data is that participants may not consent to its use. With BAM, for instance, the intention to use administrative data was a late addition (once the SDRS was ruled out), necessitating an additional process to obtain parent consent and student assent. This included visiting CYP in schools to explain what was involved. However, only a small number of boys and caregivers agreed, prohibiting meaningful analysis of crime data, so the idea was abandoned (Green et al., 2026). Some boys reacted viscerally against the idea of the evaluation team submitting their name, date of birth and postcode to the MoJ to obtain PNC data. While this request may have proven easier if bundled up with initial consent procedures (i.e., brief text on an information sheet), the fact that a very transparent and more expansive explanation elicits a largely unfavourable response raises ethical issues.

However, existing administrative data may not be appropriate for the research. This applies particularly to interventions at the universal, selective or indicated prevention levels and/or targeting younger CYP, where most participants have not engaged in crime. It is arguably even more inappropriate if the intervention and timeline for evaluating change are relatively short, leaving insufficient time to detect changes in rates of crime initiation as CYP mature. In such cases the anticipated impact on crime or violence will also often be a distal outcome in the ToC, so best measured via longer-term follow-up (not post-test). Thus, in the UK evaluation of BAM it seemed that PNC data on cautions, charges and convictions were less pertinent to an early intervention programme than expected shorter-term outcomes such as conduct problems, peer relations and school engagement (Green et al., 2026). That said, the original BAM trial in the US *did* gather administrative crime data, showing a 44% reduction in violent crime arrests (Heller et al., 2013). Contextual differences may partly explain this; US trial youth were older on average than in the UK study and over one third had been arrested prior to the trial. Further, when BAM was implemented in the UK, it was presented as a youth development programme designed to improve CYP social-emotional well-being and help CYP make effective decisions, with reduced involvement in crime a medium- or long-term intended outcome. Regardless, the ability to measure crime using administrative data is not the same as its suitability, and care is needed to consider the circumstances in which it should be done.

Partially in recognition of these challenges, there are efforts to enable researchers to track longer-term intervention impact on crime and other outcomes. In the UK, for example, the YEF evaluation data archive involves selected data from YEF-funded projects being stored in pseudonymised form,^4^ which then makes it possible to link records to government datasets such as the Department for Education’s National Pupil Database and the PNC. Researchers will be able to use the linked data to test, for example, whether a programme reduces school exclusions or crime. Data submitted by evaluators to the archive include participant identifying information (to enable pseudonymisation), the nature and amount of intervention received, socio-demographic characteristics and outcomes. Safeguards ensure the security of data, which are held by the Office of National Statistics.

The YEF archive is an impressive achievement in terms of infrastructure and addressing longstanding calls from researchers for funders and policy-makers to make it easier to measure long-term outcomes. It addresses several challenges cited earlier. In particular, it weakens the imperative to gather crime data from CYP at a time and in a way which would be premature and harmful, by giving an option to examine such data in the future when it is more relevant. This potentially allows evaluators of more preventive interventions to focus on intermediate outcomes, or mediators of crime and violence.

However, the archive is not without challenges. One is securing participant buy-in. It can be difficult to explain to participants because of the multiple steps and parties involved to ensure the data end up in the right format at the right place to be accessed by the right people. There is no guarantee that participants will agree, indeed in the BAM evaluation CYP expressed serious reservations and assent rates were low. Another issue is statistical power. Variables in the PNC measure events that are rare; if a trial has not been powered sufficiently to detect these outcomes then the archive data are of limited value, unless of course results from different trials are pooled, which in turn speaks to the value of using common measures across evaluations. There are also challenges in identifying and destroying data if participants subsequently choose to withdraw from a study because of the pseudonymisation process involved in linkage to the PNC. Finally, the archive builds in a long delay to obtaining offending outcomes. Data must first be archived, then someone has to show initiative to access it (or a funder has to commission the work), and then the study needs to happen and report. Policy-makers understandably want evidence on effectiveness promptly, so a risk of putting all eggs in the archive basket is that policy decisions are made absent useful evidence.

## Conclusion

We have explored challenges with measuring outcomes in evaluations of interventions to prevent youth crime and violence. Starting with the selection of relevant outcomes, we argue that this needs to be informed by a robust ToC. With most universal and many targeted preventive interventions the target outcomes are likely to be mediators of theorised intervention impact on crime and violence. As such, it will normally make sense during studies to focus on non-crime/violence outcomes for interventions to prevent crime and violence. This particularly makes sense when: there is good evidence that the outcomes are mediators (i.e., longitudinal studies demonstrating their causal impact on crime or violence); outcomes are to be measured over a relatively short (e.g., up to two years) timeframe (often unrealistic for detecting crime or violence outcomes); participants are younger (especially pre-adolescence); and most participants have not yet engaged in criminal activity or had contact with youth justice (partially but not entirely age-related).

Importantly, the selection of outcomes needs to consider measurement issues, otherwise there is a risk of forcing a focus on outcomes that cannot or should not be measured. To that end, we have explored alternative methods for measuring crime and violence outcomes, including CYP self-report and administrative data (see also Gomes et al., 2018). All have merits but the practical, scientific and ethical issues are difficult to overcome. Some combination of the two may be beneficial, but this does not entirely negate their respective limitations (Cornish et al., 2024). It also leaves the question about the relevance of measures *during the study* given the intervention ToC. This is where the YEF archive is potentially very useful. It frees evaluators to focus on measuring known mediators of crime and violence – in alignment with the respective intervention ToC – in the knowledge that the impact on *recorded* crime and violence outcomes can be examined later. That said, its usefulness in reality remains to be seen.

We recognise the seriousness of the harms associated with youth crime and violence, and the vital importance of research in helping to prevent these outcomes. Researchers must weigh the risks introduced by the issues described above against these longer-term societal benefits. However, where the risks of harm to study participants are considerable, and the benefits of particular outcome measurement approaches are uncertain or contested, it is not simply a matter of balancing risks and benefits, but of considering whether the ends justify the means.

In short, the ethical, scientific and practical challenges presented by self-report measures and administrative data mean that evaluators (like us) need to take great care when selecting appropriate outcomes and measuring crime/violence. We recognise the risk of naivety in expecting policymakers and funders to invest in preventive interventions for which there is no *direct* evidence of effectiveness in preventing crime or violence, so it would be unreasonable to expect evaluators to stop all use of self-reported offending measures. But the appropriate response is not to force the issue if available measurement options are illogical or harmful. Rather, we need to test alternative approaches for measuring crime and violence outcomes, including new self-report measures and measures of change, and share the learning widely, and use administrative data such as the YEF archive (whilst remaining alert to its limitations). Additionally, we need to continue, via longitudinal effectiveness studies, to build the evidence base for mediators of intervention impact on crime/violence and focus for now on measuring proximal outcomes. Such research can further inform approaches to modelling the longer-term impact on crime (and other social and health outcomes) of short-term effects on more proximal outcomes (e.g., Skarda et al., 2022).

## Footnotes

1 Annual official crime data in England and Wales are reported for the period April-March. The legal minimum age of criminal responsibility is 10 years and the youth justice system handles CYP who offend until they turn 18. A proven offence is recorded when a CYP receives a caution or a court sentence. There are different categories of offence, but they cover *inter alia* homicide, robbery, rape, assault, and the possession of knives or offensive weapons.

2 Whilst it is important to tackle bullying and cyber bullying, and we acknowledge ongoing debates on whether cyberbullying should be defined as youth crime (Zhang et al., 2022) and its association with youth offending (Lee et al., 2020), we instead concentrate on forms of antisocial behaviour and offending that are in line with our experience as evaluators.

3
https://www.gov.uk/government/publications/county-lines-exploitation.

4 Details of the archiving process, including the pseudonymisation method, can be found here: https://youthendowmentfund.org.uk/evaluation-data-archive/.
